# Effects of Different Diets on Microbiota in The Small Intestine Mucus and Weight Regulation in Rats

**DOI:** 10.1038/s41598-019-44994-7

**Published:** 2019-06-11

**Authors:** Yu Meng, Xiaojun Li, Jie Zhang, Chunlian Wang, Fanggen Lu

**Affiliations:** Departments of Gastroenterology, The Second Xiangya Hospital of Central South University, Changsha, Hunan P.R. China

**Keywords:** Dysbiosis, Obesity

## Abstract

While the microbial community of the small intestine mucus (SIM) may also play a role in human health maintenance and disease genesis, it has not been extensively profiled and whether it changes with diet is still unclear. To investigate the flora composition of SIM and the effects of diet on it, we fed SD rats for 12 weeks with standard diet (STD), high-fat diet (HFD), high-sugar diet (HSD) and high-protein diet (HPD), respectively. After 12 weeks, the rats were sacrificed, SIM and stool samples were collected, and high-throughput 16S rRNA gene sequencing was used to analyze the microbiota. We found that fecal microbiota (FM) was dominated by *Firmicutes* and *Bacteroidetes*, while in SIM, *Firmicutes* and *Proteobacteria* were the two most abundant phyla and the level of *Bacteroidetes* dramatically decreased. The microbiota diversity of SIM was less than that of feces. The community composition of SIM varied greatly with different diets, while the composition of FM altered little with different diets. The relative abundance of *Bacteroidetes* and *Allobaculum* in SIM were negatively correlated with weight gain. There was no significant correlation between FM and weight gain. In conclusion, the community profile of SIM is different from that of feces and susceptible to diet.

## Introduction

The gastrointestinal tract is chronically exposed to various antigens, mostly of bacterial origin. In the intestine, physical separation of bacteria and the epithelium is largely dependent on mucus which is primarily composed of the highly O-glycosylated mucin 2 (Muc2) secreted by goblet cells in the epithelium. The small intestine harbors a single unattached mucus layer in which different commensal bacteria reside^1,2^. Resident microbiota in the mucus layer contributes to prevent the invasion and adhesion of luminal bacteria by competing for niches and nutrition^1^. These bacteria play an active role in shaping and regulating the gut barrier^3^.

Numerous studies have shown that diet plays an important role in mediating alterations in intestinal flora composition. Studies revealing that high-fat diet (HFD) was responsible for the dysbiosis with decrease in Gram-positive bacteria in the gut lumen^4,5^. Ingestion of high-fiber diet could raise the levels of *Lactobacillus*, *Bifidobacterium*, *E. coli*^6^, and bacteria producing short-chain fatty acids^7^. Rats fed with proteins from beef, pork and fish had a higher level of *Firmicutes*, while rats fed with casein and soy protein had an increase in the abundance of *Bacteroidetes*^8^. During infant period, the microbiota of breast-fed infants is more dominated by *Bifidobacterium* and *Bacteroides* compared with formula-fed infants^9^. The intestinal bacterial structure responds differently to different dietary composition. So far, the majority of microflora analysis results are based on samples taken from the fecal contents or mucosal tissues of the large intestine, especially fecal samples. These samples are often used because they are easily collected. However, the small intestine mucus (SIM) samples are rarely used to investigate the microflora.

The different regions of the intestine harbor distinct bacterial communities^10^. In humans, *Firmicute*s and *Actinobacteria* account for the predominant phyla in duodenal samples, while *Bacteroidetes* are not detected^11^. *Firmicutes* and *Bacteroidetes* have been identified as the major phyla in the small intestine contents of mice^12^. This distribution of phyla is distinctly different from the phyla found in both human and mouse feces, which are dominated by *Firmicutes* and *Bacteroidetes*^13^. Also, it has been found that bacterial community in the mucus differs from that in stool and intestinal lumen^14,15^, and mucus-resident microbiota varies most based on location^15^. To date, the bacterial community in SIM has not been extensively profiled and whether the microbiota shifts with diets is largely unknown.

Available data indicate that intestinal microbes may affect goblet cell and the mucus layer directly. Changes in goblet cell and in the chemical composition of intestinal mucus are detected in response to diets and alterations of the normal microbiota. The results of a study made by Sharma R *et al*. (1995), in which germ-free and conventionally maintained rats were fed two different diets and a group of rats born germ-free was inoculated with human flora, showed both rat and human floras reduced the number of cells containing mucins in the small intestine of rats fed on a purified diet, and feeding a commercial diet reduced the volume density of cells containing mucins in the jejunum of conventional rats and the staining density of mucins in the germ-free rats^16,17^. A study on parenterally and orally fed piglets found an indicator of localized inflammation and goblet cell numbers in the ileum of piglets with total parenteral nutrition^18^. Another study provided evidence that the dietary composition, microbial flora, as well as the interactions between the dietary constituents and microbial flora changed the mucosal architecture and the mucus composition^19^. These findings demonstrate that the dietary changes and microbial populations are influential in modifying the amount and proportion of mucins in the small intestine. How the small intestinal mucosa of rats adapts to unbalanced diets, including HFD, high-sugar diet (HSD) and high-protein diet (HPD) remains to be understood.

In this study, we investigated (i) the microbial community of in SIM and feces of rats fed various diets by using 16S rRNA sequencing, (ii) the relationship between changes in microbial composition and weight gain in rats, and (iii) the effects of various diets on the small intestinal mucosa.

## Results

### Differences of weight gain in rats fed on different diets

**Figure 1:**
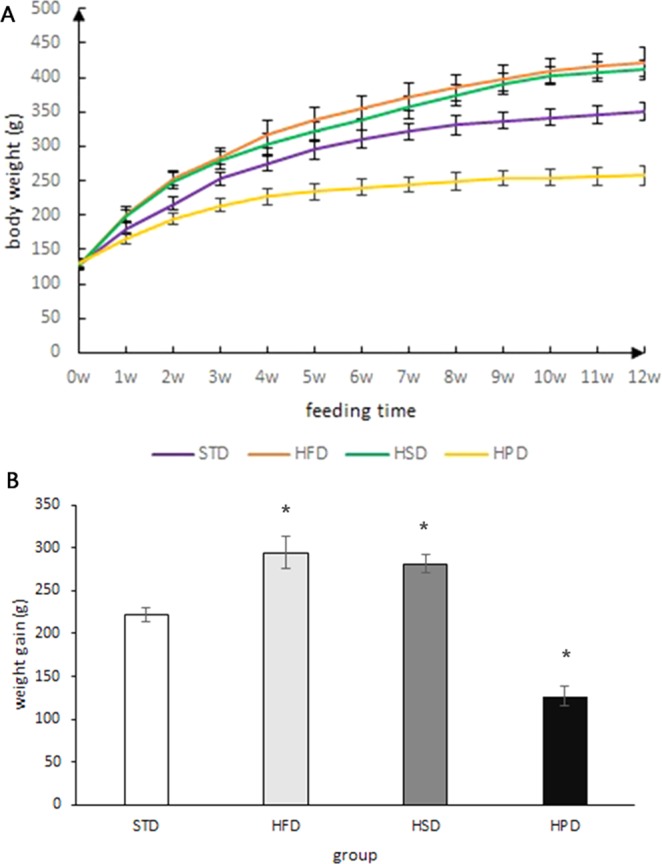
(**A**) Weight changes in rats fed on different diets at different time points. (**B**) Weight gain in rats fed on different diets for 12 weeks. (*vs STD group, P < 0.05). There was a significant increase in the rats fed HFD or HSD compared with those fed STD, but there was no significant difference in weight gain between the rats fed HFD and those fed HSD. The rats fed HPD gained the least weight.

The initial body weight of the rats fed standard diet (STD), HFD, HSD or HPD was 129.1 ± 8.14, 127.43 ± 5.40,129.6 ± 6.69 and 130.1 ± 5.24 g, respectively. After 12-week feeding, the body weight of rats in each group increased to 350.6 ± 13.03, 422.1 ± 20.58, 410.9 ± 14.09, and 257.2 ± 13.01 g, respectively. The rats fed HFD or HSD gained significantly more weight than those fed STD (P < 0.01), but there was no significant difference in weight gain between the rats fed HFD and those fed HSD (P = 0.198). The weight gain of the HPD-fed rats was significantly less than the STD-fed rats. (P = 0.000) (Fig. 1).

### The composition of microbiota in SIM and feces of rats fed on STD

**Figure 2:**
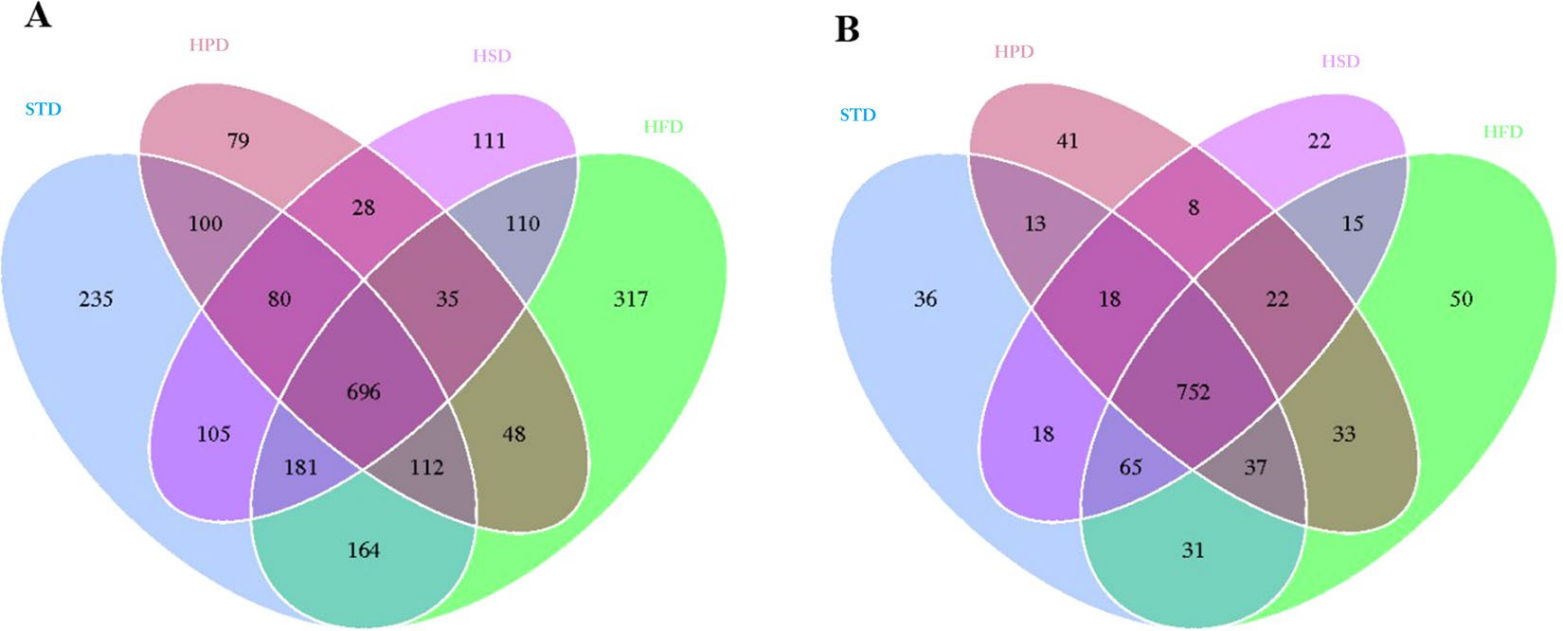
Venn Diagrams based on the shared and unique OTUs. (**A**) The shared OTUs among the SIM samples of different groups and the unique OTUs in the SIM samples of each group. (**B**) The shared OTUs among the fecal samples of different groups and the unique OTUs in the fecal samples of each group.

320564 usable raw reads were obtained from 28 SIM samples and paired fecal samples. We used the Venn diagrams to show the interrelationship of OTUs in the fecal samples and SIM samples among different groups (Fig. 2A,B). There were 696 OTUs shared in the SIM samples of each group and the number of OTUs unique to the STD group, HPD group, HSD group and HFD group was 235, 79, 111 and 317, respectively. In comparison, 752 OTUs were shared in the fecal samples of each group and there were 36, 41, 22 and 50 unique OTUs in the fecal samples of STD group, HPD group, HSD group and HFD group, respectively.

**Figure 3:**
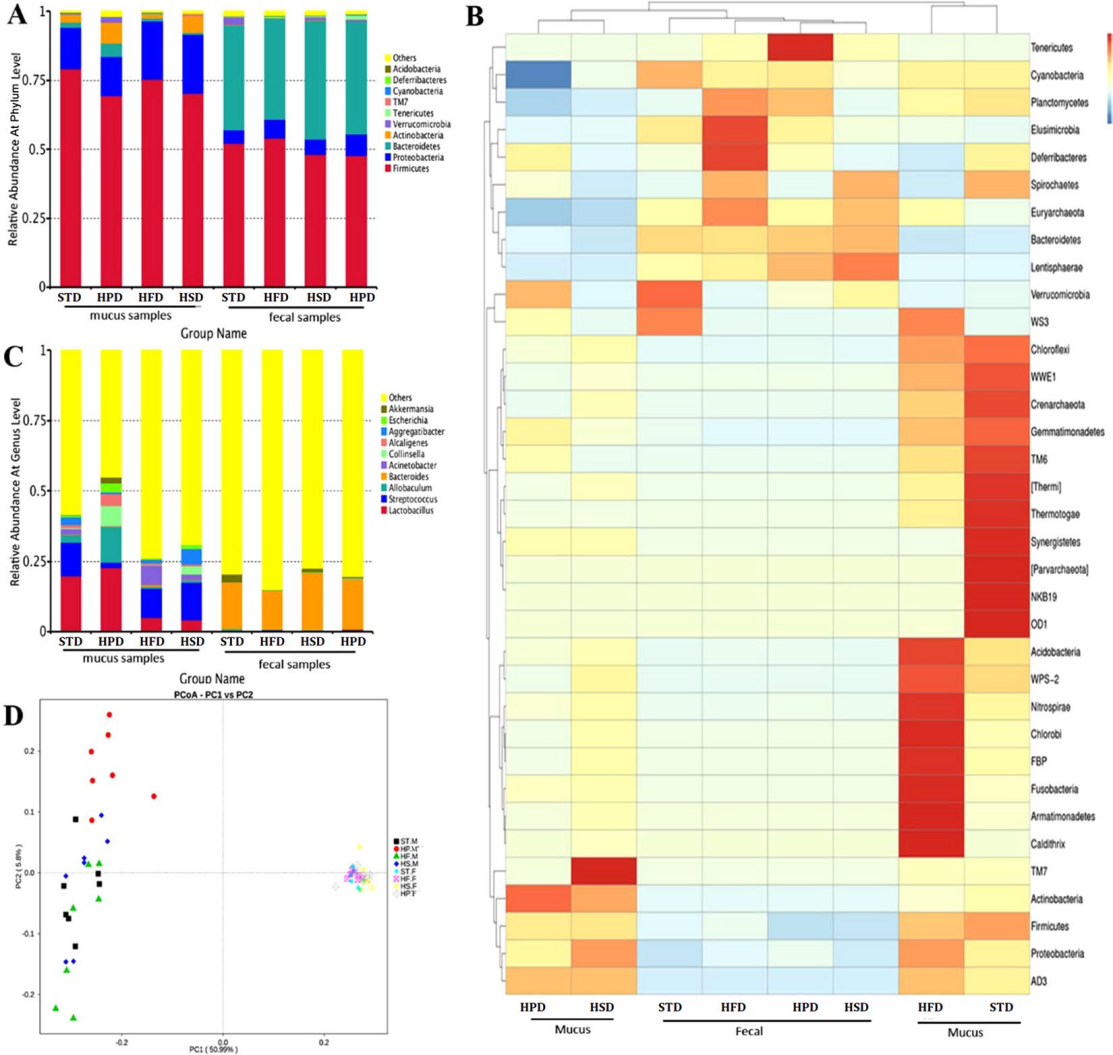
(**A**) Relative abundance of microflora at phylum level in SIM and fecal samples of each group. *Firmicutes* and *Proteobacteria* were the most abundant phyla in SIM of each group, while *Bacteroidetes* and *Firmicutes* were the major phyla in fecal samples of each group. *Actinobacteria* was the third phylum in SIM, but not found in fecal samples. (**B**) Heatmap of the predominant genera identified in SIM and fecal samples of each group. (**C**) Relative abundance of microflora at genus level in SIM and fecal samples of each group. *Lactobacillus*, *Streptococus*, and *Allobaculum* were the dominant genera in SIM; *Bacteroides* was the major genus in fecal samples. (**D**) PCA displayed the distribution of corresponding points of SIM samples and fecal samples of each group.

To address the top ten relative abundance of microbial group, the taxonomic classification is performed at phylum, class, order, family, genus and species level, respectively. The microbiota analysis of rats fed STD revealed that there was a dramatic decrease in the relative abundance of *Bacteroidetes* in SIM samples compared to fecal samples (2.04 ± 1.57% vs 38.11 ± 5.14%, P < 0.01). The relative abundance of *Firmicutes*, *Proteobacteria* and *Actinobacteria* in SIM samples was significantly higher than in fecal samples (79.20 ± 4.25% vs 52.17 ± 3.12%, P < 0.01; 14.94 ± 3.73% vs 4.79 ± 1.00%, P < 0.01; 2.67 ± 0.96% vs 0.14 ± 0.08%, P < 0.01). Verrumcomicrobia were detected only in fecal samples. These results suggested that most of the sequences in SIM samples belonged to *Firmicutes* and *Proteobacteria*, accounting for more than 90% of abundance at phylum level, while the rest mainly distributed in *Actinobacteria* and *Bacteroidetes*. Fecal microbiota (FM) was dominated by *Firmicutes* and *Bacteroidetes* (Fig. 3A). Also, there were great differences in the microflora composition between SIM samples and fecal samples at the genus level. *Lactobacillus*, *Streptococus* and *Allobaculum* were dominant genera in SIM not in fecal samples (19.79 ± 20.85% vs 0.14 ± 0.03%, P < 0.01; 11.84 ± 7.06% vs 0.22 ± 0.14%, P < 0.01; and 2.79 ± 1.63% vs 0.70 ± 1.33%, P < 0.01). *Bacteroides* was found mainly in fecal samples (Fig. 3B,C).

### The composition of microbiota in SIM and feces of rats fed on unbalanced diets

In addition to the rats fed STD, the composition of SIM microflora was also different from that of FM in the rats fed unbalanced diets. *Firmicutes*, *Proteobacteria* and *Actinobacteria* were the top three abundant phyla in SIM samples of all the unbalanced diet groups, while in fecal samples of these groups, *Bacteroidetes* and *Firmicutes* were the major phyla. In the rats fed on HFD, the abundance of *Firmicutes* and *Actinobacteria* in SIM was higher than that in feces (75.39 ± 18.21% vs 47.69 ± 5.60%, 1.55 ± 0.57% vs 0.08 ± 0.04%, respectively, P < 0.05), and at the genus level, the abundance of *Lactobacillus* and *Streptococcus* was also higher in SIM than that in feces (4.98 ± 3.37% vs 0.41 ± 0.16%, 10.55 ± 7.61% vs 0.18 ± 0.06%, respectively, P < 0.05). In the rats fed on HSD, higher abundance of *Proteobacteria*, *Actinobacteria*, *Lactobacillus*, *Streptococcus*, *Allobaculum*, and lower level of *Bacteroidetes* were present in SIM than that in feces (P < 0.05).

The community composition in SIM samples had different alterations with different diets. In comparison with the rats fed STD, the rats fed HFD or HSD had a decreased abundance of *Bacteroidetes* in SIM (P < 0.05) and the rats fed HPD had an increased abundance (4.70% ± 6.29% vs 2.03% ± 1.57%, P < 0.05). The relative abundance of *Actinobacteria* decreased in SIM samples of HFD group (1.55% ± 0.57%vs 2.68 ± 0.96%, P < 0.05), and rose in HSD group and HPD group (P < 0.05). The analysis results of FM showed that the rats fed HPD had fewer abundance of *Firmicutes* than those fed STD (48.21% ± 7.92% vs 54.05% ± 5.44%, P < 0.05); Higher level of *Proteobacteria* was detected in the rats fed HPD or HFD compared with those fed STD (P < 0.05); there was no significant difference in the proportion of *Proteobacteria* between HSD-fed rats and STD-fed rats (P > 0.05); the rats fed HSD had a higher abundance of *Bacteroidetes* than those fed STD (43.14% ± 6.71% vs 38.12% ± 5.14%, P < 0.05).

At the genus level, the composition of flora in SIM samples also shifted with unbalanced diets. The microbiota analysis on SIM samples revealed that there was a dramatic decrease in the relative abundance of *Streptococcus* in the rats fed HPD compared to those fed STD(1.93% ± 2.18% vs 11.84% ± 7.06%, P < 0.05); there was no significant difference in the level of *Streptococcus* between the rats fed HFD or HSD and those fed STD (P > 0.05); the abundance of *Lactobacillus* was higher in HPD group than STD group (22.71% ± 15.47% vs 19.79% ± 20.85%, P < 0.05) and decreased in HFD group and HSD group compared with STD group (P < 0.05); the level of *Allobaculum* also increased in HPD group and decreased in HFD group and HSD group. The relative abundance of *Acinetobacter* rose in HFD group and decreased in HPD group. In contrast, the community profile in fecal samples did not change notably with the diets, either at the phylum level or at the genus level, except that the abundance of *Verrumcomicrobia* and *Akkemansia* decreased in various unbalanced diet groups. (Fig. 3A–C)

### Alpha diversity and beta diversity of microbiota in SIM and feces

**Figure 4:**
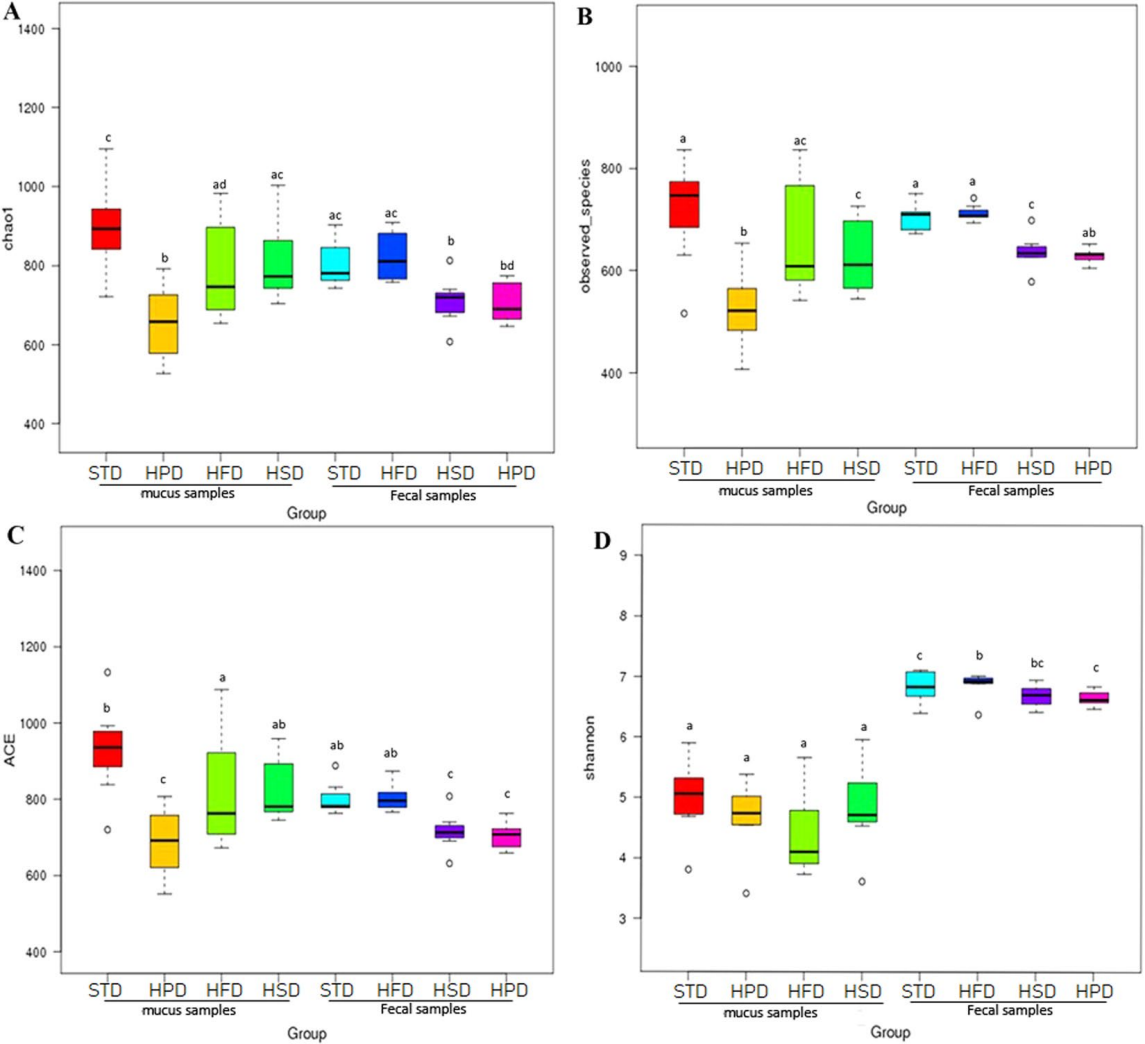
Box plot of alpha diversity showing differences between each other group. (**A**) Chao1 index (**B**) The observed species (**C**) ACE index (**D**) Shannon index. The letters a, b, c and d are used to clarify whether the difference between any pair of groups calculated by Wilcoxon analysis was statistically significant (p < 0.05), and there was a significant difference in flora diversity between the two groups sharing no common letter markers.

The microbial alpha diversity was estimated based on the originally observed count values prior to any pre-processing, Chao1 index and ACE index were used as measures of community richness. The Shannon index was used as a measure of taxa richness and evenness. Chao1 indexes of microflora in SIM and fecal samples of STD, HFD, HSD and HPD group were as follows: SIM 897.05 ± 117.76, 793.51 ± 132.66, 812.99 ± 104.84, and 655.25 ± 102.63; FM 806.31 ± 60.16, 824.72 ± 65.19, 709.12 ± 63.22, and 707.73 ± 53.30. ACE indexes of SIM microflora were different from that of FM in each group (P < 0.05, Mann-Whitney test). In each group, Shannon indexes of SIM microflora were significantly less than that of FM (STD 4.98 ± 0.66 vs 6.84 ± 0.28, HFD 4.41 ± 0.70 vs 6.87 ± 0.22, HFD 4.86 ± 0.77 vs 6.69 ± 0.19 and HPD 4.67 ± 0.64 vs 6.66 ± 0.13, P < 0.05, respectively) (Fig. 4), indicating microbiota diversity of SIM was lower than FM.

**Figure 5:**
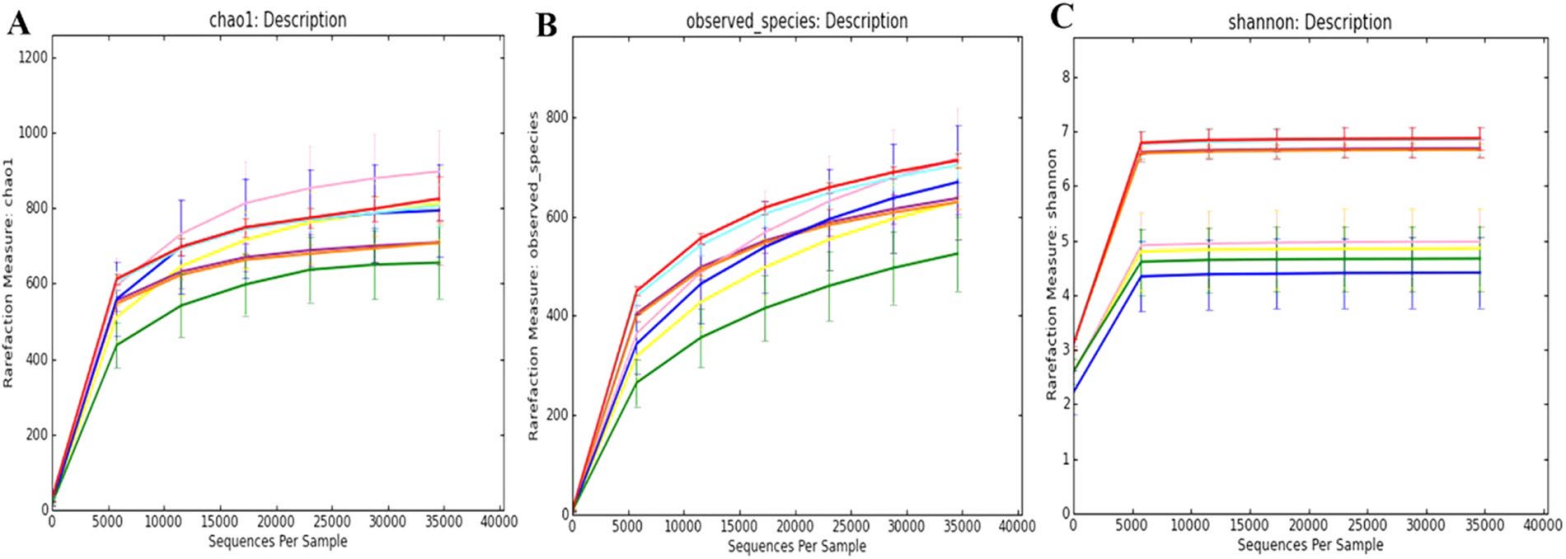
Rarefaction curves which was used to judge whether further sampling would likely yield additional taxa by whether the curve reached a plateau value. (**A**) Rarefaction curves of Chao 1 index. (**B**) Rarefaction curves of observed species. (**C**) Rarefaction curves of Shannon index.

Moreover, Chao1 index, the observed species and Shannon index reached saturation, and the rarefaction curve of each sample also entered the plateau phase (Fig. 5), indicating the sequencing method was appropriate to evaluate the microbial diversity in the present study. Wilcoxon analysis revealed that in the rats fed STD, the community richness in SIM did not significantly differ from fecal community richness, while the community diversity in SIM was significantly less than fecal community diversity. The observed species, Chao1 index, ACE index and Shannon index were shown in Table 1. The results unveiled the diversity of microbiota in SIM samples was greater at all levels than that of FM.

**Table 1:** Alpha diversity.

Group	Observed Species(x ± SD)	Chao1 (x ± SD)	Shannon (x ± SD)	ACE (x ± SD)
STDG-M	716.714 ± 108.552	897.047 ± 117.761	4.978 ± 0.664	930.605 ± 128.437
HFDG-M	668.714 ± 123.302	793.514 ± 132.661	4.411 ± 0.699	826.018 ± 155.618
HSDG-M	629.429 ± 77.743	812.994 ± 104.839	4.855 ± 0.770	829.541 ± 85.448
HPDG-M	524.857 ± 81.321	655.250 ± 102.634	4.668 ± 0.643	686.901 ± 98.776
STDG-F	702.857 ± 28.145	806.307 ± 60.156	6.842 ± 0.277	802.140 ± 43.780
HFDG-F	712.429 ± 16.339	824.718 ± 65.188	6.870 ± 0.223	804.078 ± 38.419
HSDG-F	636.429 ± 35.804	709.124 ± 63.220	6.688 ± 0.189	715.710 ± 53.080
HPDG-F	628.429 ± 14.831	707.733 ± 53.300	6.659 ± 0.127	703.627 ± 36.135

STDG: standard chow group, HFDG: High-fat diet group, HSDG: High-sugar diet group, HPDG: High-protein diet group, M: the small intestine mucus samples, F: the fecal samples.

Beta diversity was analyzed using Principle component analysis (PCA), as was shown in Fig. 3D. The distribution of points corresponding to SIM samples was discrete among different dietary groups, indicating the community composition of SIM varied greatly with different diets. Also, the distribution of corresponding points of fecal samples in each group tended to cluster, which meant the composition of FM altered little with different diets. Furthermore, there were apparent distances between corresponding points of SIM samples and those of fecal samples in each group, which suggested there were notable differences in microbial composition between SIM samples and fecal samples. These results demonstrated that microbiota of SIM, being different from FM, was susceptible to diet.

To compare the composition of SIM microbiota with that of FM, and to investigate the community structures in response to different diets, Anosim and MPRR analysis were performed. Significant differences were observed between every two groups (P < 0.05), and there were significant differences in the microbial structure between SIM and fecal samples of each group (P < 0.05) (Tables 2 and 3).

**Table 2:** Anosim analysis.

Group	R-value	P-value
STD.F-STD.M	1	0.001
HFD.F-HFD.M	1	0.002
HSD.M-HSD.F	1	0.001
HPD.M-HPD.F	1	0.001
SDT.M-HPD.M	0.3168	0.004
STD.M-HFD.M	0.2012	0.022
STD.M-HSD.M	0.2536	0.017
HFD.M-HPD.M	0.6628	0.001
HFD.M-HSD.M	0.3528	0.008
HSD.M-HPD.M	0.6433	0.001
STD.F-HPD.F	0.5967	0.001
STD.F-HFD.F	0.1987	0.009
STD.F-HSD.F	0.1395	0.054
HFD.F-HSD.F	0.691	0.001
HFD.F-HPD.F	0.6142	0.001
HSD.F-HPD.F	0.553	0.004

R value was ranged from −1 to 1, R. > 0, significant differences between groups, R < 0, significant differences between samples within the group. P < 0.05 was considered statistically significant.

**Table 3:** MRPP analysis.

Group	A	Observed-delta	Expected-delta	Significance
STD.F-STD.M	0.3604	0.4437	0.6937	0.001
HFD.F-HFD.M	0.4555	0.3661	0.6723	0.003
HPD.F-HPD.M	0.3384	0.4692	0.7092	0.001
HSD.F-HSD.M	0.4112	0.4067	0.6908	0.002
STD.F-HPD.F	0.08692	0.3304	0.3618	0.002
STD.F-HFD.F	0.03949	0.3354	0.3492	0.002
STD.F-HSD.F	0.01504	0.3276	0.3326	0.106
HFD.F-HSD.F	0.07977	0.3165	0.3440	0.002
HFD.F-HPD.F	0.05896	0.3193	0.3393	0.001
HPD.F-HSD.F	0.06054	0.3115	0.3316	0.002
STD.M-HPD.M	0.06111	0.5825	0.6204	0.006
STD.M-HFD.M	0.0594	0.4744	0.5043	0.024
STD.M-HSD.M	0.04877	0.5228	0.5496	0.046
HPD.M-HSD.M	0.1274	0.5643	0.6467	0.002
HPD.M-HFD.M	0.1786	0.516	0.6283	0.001
HFD.M-HSD.M	0.06402	0.4562	0.4874	0.019

Observe Delta reflects the variety within the group. Expect delta represents variety between groups. A > 0, significant differences between groups. A < 0, significant differences within the group. P < 0.05 was considered statistically significant.

### Correlation analysis between weight gain and gut microbiota

**Figure 6:**
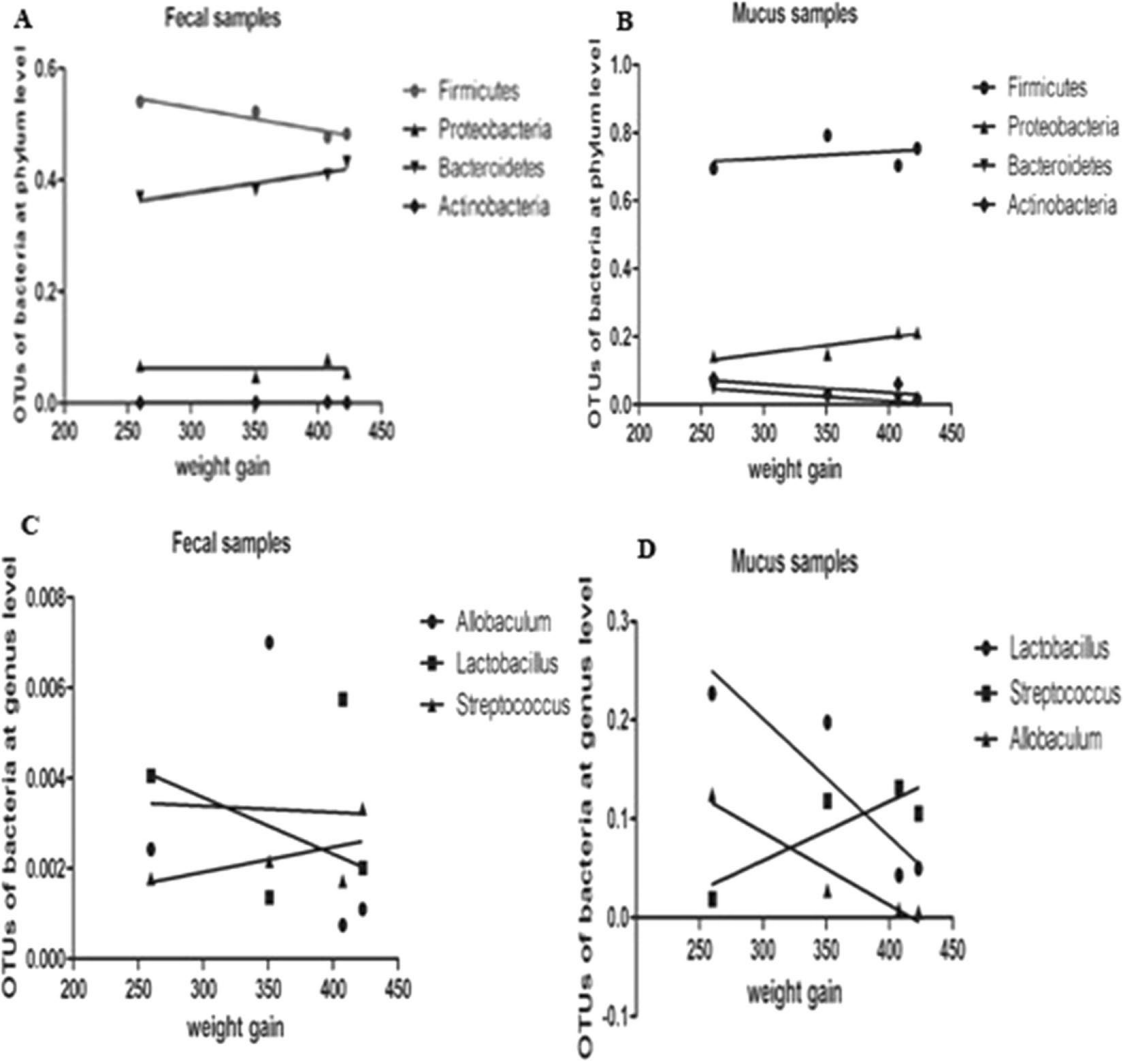
Correlation analysis between gut microbiota and weight gain. (**A**) The abundance of *Bacteroidetes*, *Proteobacteria*, *Firmicutes* and *Actinobacteria* in fecal samples had no significant correlation with weight gain. (**B**) *Bacteroidetes* in SIM samples was negatively correlated with weight gain (P = 0.04, r = −0.46). (**C**) The abundance of *Allobaculum*, *Streptococcus* and *Lactobacillus* in fecal samples was not correlated with weight gain. (**D**) In SIM samples, the abundance of *Allobaculum* was negatively correlated with weight gain (P = 0.033, r = −0.65), while *Streptococcus* and *Lactobacillus* were not significantly correlated with weight gain.

Correlation analysis was used to explore the relationship between weight gain and microbiota in the SIM and feces. The bacterial flora analysis among various groups revealed the relative abundance of *Bacteroidetes* in SIM samples decreased in the HFD-fed rats and HSD-fed rats fed, and increased in the rats fed HPD. Correlation analysis further unveiled a significant negative correlation between the relative abundance of *Bacteroidetes* in SIM samples and weight gain (P = 0.04, r = −0.46). The relative abundance of *Firmicutes*, *Proteobacteria* and *Actinobacteria* in SIM samples was not significantly correlated with weight gain (P = 0.21, P = 0.06, P = 0.26, respectively) (Fig. 6A). No significant correlation was found between the relative abundance of *Bacteroidetes*, *Firmicutes*, *Proteobacteria* and *Actinobacteria* in fecal samples and weight gain(P > 0.05) (Fig. 6B). At the genus level, the relative abundance of *Allobaculum* in SIM decreased with the increase in body weight of the rats. Further correlation analysis found that *Allobaculum* were negatively correlated with weight gain (P = 0.033, r = −0.65). *Streptococcus* and *Lactobacillus* were not significantly correlated with weight gain (Fig. 6).

### Effects of various diets on the goblet cell number in the small intestinal epithelium of rats

**Figure 7:**
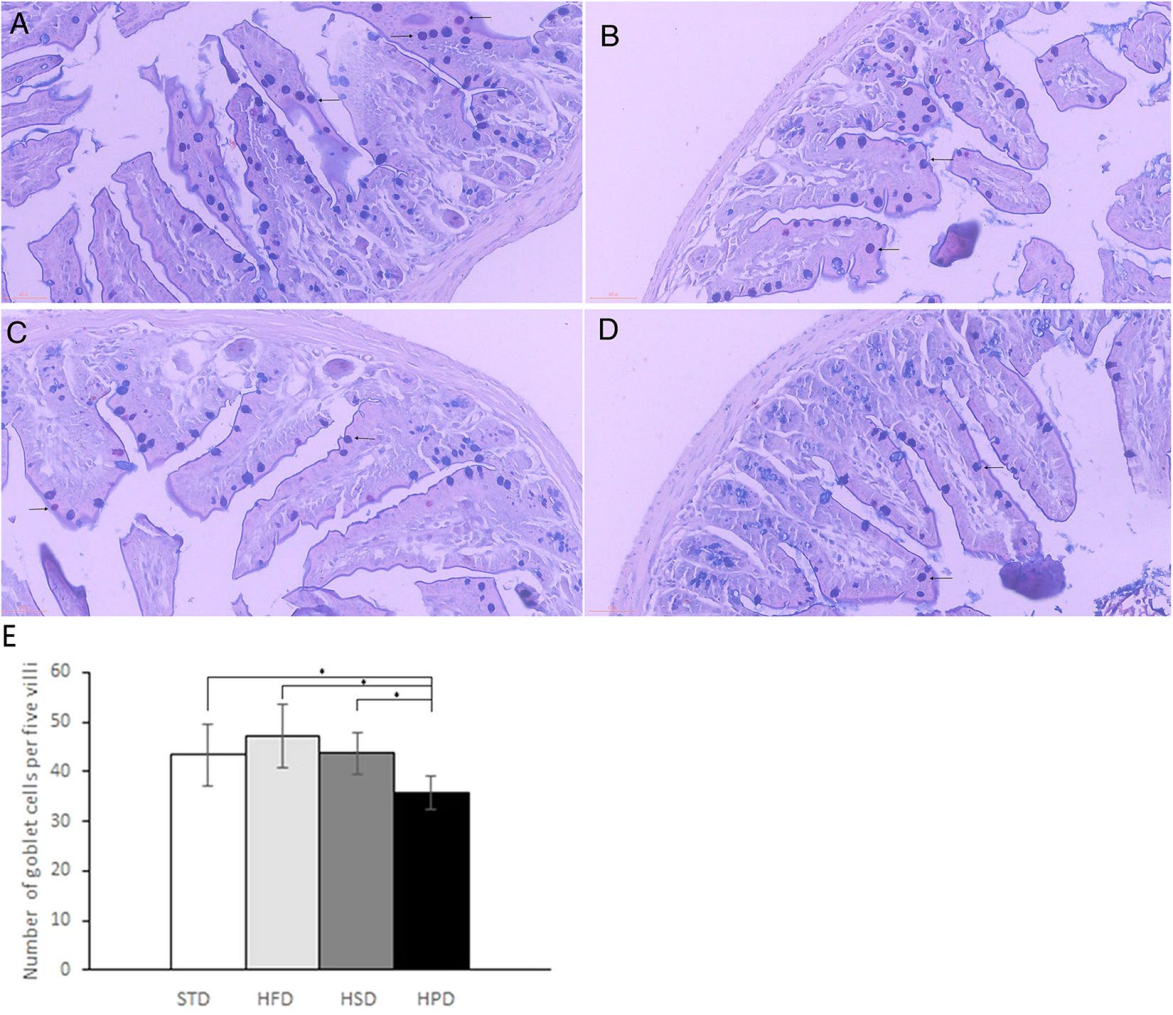
Representative images of AB-PAS staining of ileum tissues of STD group (**A**), HFD group (**B**), HSD group (**C**) and HPD group (**D**). (**E**) Number of goblet cells per 5 villi in the small intestinal epithelium. The mucus-positive cell number was significantly lower in HPD group than STD, HFD and HSD group, *P < 0.05.

To understand the effects of various diets on the SIM structure, AB/PAS-positive cells in the epithelium were counted (Fig. 7A–D). No significant difference in the number of mucus-positive cells was observed between HFD group and STD group (47.2 ± 6.3 vs 43.3 ± 6.2 cells per 5 villi, P = 0.357) as well as between HFD and HSD group (47.2 ± 6.3 vs 43.7 ± 4.3 cells per 5 villi, P = 0.332). There was also no significant difference between HSD and STD group (P = 0.924). The mucus-positive cell number in HPD group (35.7 ± 3.2 cells per 5 villi) was significantly lower than that in STD group, HFD group and HSD group (P < 0.05) (Fig. 7E). Goblet cell number in villous epithelium of small intestine revealed that both acidic and neutral mucins in small intestinal epithelium were less abundant in response to HPD feeding than to STD feeding, and had no significant change in respond to HFD and HSD compared to STD.

## Discussion

Gut microbiota plays profound roles in host health and disease. Considerable research has focused on understanding the communities in the contents and mucosal tissue of the large intestine, especially FM^5,20–24^. Some information is also available about the microflora composition in the contents of small intestine^20^. However, there are few studies exploring the community profile in SIM. In this study, we investigated and compared the microbial structure of SIM and feces. The results showed that the microflora composition of SIM was markedly different from that of feces; *Firmicutes* and *Proteobacteria* were the two most abundant phyla in SIM, accounting for more than 90% relative abundance of the community, and the third abundant phylum was *Actinobacteria*; FM was dominated by *Firmicutes*, *Bacteroidetes* and *Proteobacteria*. It has been shown that *Bacteroidetes*, *Firmicutes* and *Proteobacteria* were the major phyla in the small intestine contents as well as in the cecal and colonic contents; anaerobic bacteria, including *Alkaliphilus*, *Butyricimonas*, *Clostridium* and *Parabacteroides spp*, accounted for the most abundant genera in the small intestine contents^20^. One research investigating gut microbiota in rhesus macaques has also found that the community in fecal samples could not stand for that in the small intestine contents, and in mucosal samples, facultatively anaerobic clades, such as *Helicobacter* in the large intestine and *Pasteurella* in the small intestine were more abundant^15^. In this study, we also found that the predominant genera in SIM, including *Lactobacillus*, *Streptococcus* and *Allobaculum*, belonged to facultative anaerobes, while the relative abundance of these bacteria in feces was very low. This may be attributed to the fact that the mucous layer lies on top of the epithelial surface, which may contribute to the diffusion of oxygen from the blood to the mucus resulting in the higher oxygen content in the mucous layer compared to the gut lumen. Moreover, we found the community diversity of SIM was less than that of feces, which was also confirmed by the previous study^25^. ß diversity analysis also suggested there were notable differences in microbial composition between SIM samples and fecal samples. All the findings indicated that there is a characteristic microflora community in SIM, not only different from that in colonic contents but also from that in small intestine contents.

The microbial community profile of SIM is different from that of small intestine contents and feces, which may be due to the environment where the microflora survives. Intestinal contents mainly come from food and provide nutrition for the bacteria in the contents. Microflora composition in intestinal contents is influenced by diet. For example, the composition of fecal flora in Western-style diet population differs from that in high-fiber diet population^22^. In comparison, the microflora resident in the mucus layer is mainly nourished by mucin. Intestinal contents are able to contact with the mucus with the flow along the gastrointestinal tract, resulting in bacteria in the mucus can be inoculated into the luminal contents. In this way, the microbial community in the intestinal contents comprises bacteria coming from diet and SIM, and fecal microbiota may include bacteria in the mucus along the whole gastrointestinal tract.

So far, it is not clear yet whether diet has impacts on the community profile in SIM. We found the abundance of *Bacteroidetes* and *Actinobacteria* in SIM decreased in the rats fed HFD; HSD-fed rats had increased level of *Actinobacteria* and decreased level of *Bacteroidetes*; increased abundance of *Actinobacteria* and *Bacteroidetes* was present in the rats fed HPD; the community richness of SIM in the rats fed on HFD or HPD increased. These results suggested that different dietary structure lead to different alterations in the composition of microflora in SIM. Furthermore, higher level of *Proteobacteria* and lower abundance of *Firmicutes* were found in SIM of rats fed HPD, and a higher abundance of *Bacteroidetes* and *Proteobacteria* were present in SIM of HSD-fed rats and HFD-fed rats, respectively. Thus, it can be seen that the response of SIM microflora to a same diet is distinct from that of colonic contents microflora. The result of ß diversity displayed the composition of SIM microbes varied greatly with different diets while the alterations of FM composition was not obvious with diets, demonstrating that microbiota of SIM was susceptible to diet compared to FM. Therefore, contrary to the more stable FM^26,27^, the SIM microbiota most likely reflect the subject dietary variation. However, there is limited information about the effects of different diets on SIM flora, and the exact mechanism of the effects of various unbalanced diets on the bacterial flora resident in SIM is still unclear. Studies have shown that undigested protein and peptide can be fermented by colonic bacteria into ammonia, indole, phenol and hydrogen sulfide^28^. These substances not only change the pH value of colonic contents but also are harmful to some bacteria, which contributes to the alterations in the composition of microbiota in colonic contents^29,30^. Since the small intestine is the main part responsible for digestion and absorption due to its function and anatomical structure, further research is needed to study whether the different effects of various diets on the microbiota structure in SIM are also performed by the metabolic products derived from the dietary ingredients.

We observed more weight was gained in the rats fed on HFD or HSD while less was gained in the rats fed on HPD. Correlation between fecal microbiota and weight gain has been found in other studies. In this study, we found a negative correlation between the abundance of *Bacteroidetes* in SIM and the rats weight gain, and the level of *Firmicutes* and *Proteobacteria* in SIM had no correlation with weight gain. This finding differed from the results on association between fecal flora and weight gain. Previous studies have found the abundance of *Bacteroidetes* decreased in obese individuals^31,32^, which is consistent with our results that the HFD -fed or HSD-fed rats with more weight gain had lower abundance of *Bacteroides* in SIM and the HPD -fed rats with less weight gain had higher level of *Bacteroides* in SIM. In addition, the bacteria associated with weight gain were different among the rats fed different diets. The level of *Actinobacteria* in SIM was positively correlated with weight gain in the rats fed HPD and negatively correlated in the rats fed HFD. There was no correlation between fecal *Actinobacteria* abundance and weight gain of the rats in each group. These results further indicate the association between the bacterial flora in SIM and weight changes is distinct from that between FM and weight change. Furthermore, we found the number of goblet cells significantly decreased in the rats fed HPD. Intestinal mucus is primarily composed of Muc 2, which is secreted by goblet cells in the epithelium^1^. Decreased number of goblet cells results in a decrease in mucin secretion, which in turn reduces the thickness of the mucus layer. Further study may be needed to make clear whether HPD plays a role in the structural changes of microflora in SIM by affecting goblet cells.

In conclusion, microbial composition in SIM is distinct from that in feces and susceptible to different dietary composition. A decrease in the number of goblet cells may be a contributor to alterations in microflora composition in SIM associated with HPD. *Bacteroidetes* and *Allobaculum* in SIM was negatively correlated with weight gain. Further study may be needed to make clear whether HPD plays a role in the structural changes of microflora in SIM by affecting goblet cells.

## Methods

### Animals

4-week-old female Sprague-Dawley (SD) rats were purchased from housed in a specific-pathogen-free (SPF) environment in the Laboratory Animal Center of People’s Hospital of Hunan province (Changsha, China) in a 12-hour light/dark cycle. After a 1-week adaptation period, the rats were randomly assigned to four groups and shifted to one of the following sterile diets: STD, HFD, HPD or HSD for 12 weeks. The study design was shown in Fig. S3. The diets were purchased from Huafukang Biotechnology Co. Ltd (Beijing, China) and the compositions of diet were mentioned in Table 4.

**Table 4:** Dietary Composition.

Feed	Standard	High protein	High sugar	High fat
Casein	200	619.9	200	271.9
Corn starch	547	122.6	75.5	0
Dextrin	0	0	0	133.2
Sucrose	100	99.3	571	135.9
Soya oil	70	69.5	70	70
Lard	0	0	0	275.2
Cellulose	50	49.6	50	68
Pectin	0	0	0	0
Minerals	35	35	35	35
Vitamins	10	10	10	10
L- cystine	3	9.3	3	4.1
Choline	2.5	2.5	2.5	4.2
TBHQ	0.014	0.014	0.014	0.07
Total	1017.514	1017.714	1017.014	1007.57

### Sampling

Groups of rats (n = 7) were housed in individual ventilated cages, and sacrificed at 17-week-old of age. Small intestinal mucus samples were collected by aspirating and scraping from the duodenum to the terminal ileum into sterile EP tubes on the benchtop. Feces were collected with sterile forceps from the terminal portion of the colon into sterile tubes. Immediately after collection, the tubes were flash-frozen in liquid nitrogen and were stored at −80 °C until processed^20^. The small intestines were removed and the ileum tissues were fixed in paraformaldehyde and embedded in paraffin as previously described^33^.

### DNA extraction

Bacterial genomic DNA was extracted from the fecal and SIM samples using cetyltrimethylammoniumbromide (CTAB) method. The steps of DNA extraction were as follows: The sample was added with 2% w/v CTAB (HiMedia, India) containing freshly prepared lysozyme followed by incubation at 65 °C for 30 min, then phenol (pH 8.0)/chloroform/isoamyl alcohol (25:24:1) was added. Whole content was vortexed and centrifuged at 12,000 rpm/10 min, 4 °C, as described previously^34^. An equal amount of chloroform: isoamyl alcohol (24:1) was added to the liquid fraction, mixed thoroughly and incubated at room temperature for 10 min. Whole content was centrifuged at 12,000 rpm/10 min, 4 °C. Isopropanol (Sigma-Aldrich) were added into the supernatant and incubated for 6 h at −20 °C. Pellet was seen visually after the incubation. Entire content was centrifuged at 12,000 rpm/10 min, 4 °C. Pellet obtained was washed thrice with 70% ethanol (Fisher Scientific) by centrifugation at 12,000 rpm/10 min, 4 °C. Ethanol was removed completely after washings, and DNA pellet was dried without heating, Double distilled water was added to DNA pellet at an elevated temperature of 55 °C for 10 min. DNA concentration and purity were monitored on 1% agarose gels. DNA was stored at −20 °C.

### Bacterial metagenomes and 16S rRNA sequencing

Libraries and sequencing were carried out by the novogene Biotechnology Center at Beijing (China). Amplified products of the V4 region of the 16S rRNA gene originating from the BAC fraction were sequenced with IlluminaHiSeq2500 PE250 (Noher, Beijing, China) using barcoded 515 F (GTGCCAGCMGCCGCGGTAA) and 806 R (GGACTACHVGGGTWTCTAAT) primers, and the average read length was 250 bp. All PCR reactions were carried out with Phusion® High-Fidelity PCR Master Mix (New England Biolabs, Ipswich, USA). SYB green contained 1ψloading buffer (Solarbio, Beijing, China), and PCR products were resolved on 2% agarose gel electrophoresis. Band of 400–450 bp was chosen for further experiment. The composition of a PCR reaction was as follows: 0.3 μM primers, 0.3 mM dNTPs, 0.5 U polymerase enzyme, and 50 ng DNA template. The PCR program consisted of the following steps: initial denaturation at 98 °C for 1 min, 30 cycles at 98 °C for 10 s, 50 °C for 30 s, and extension at 72 °C for 60 s.

Amplicons were sequenced on a HiSeqIllumina platform (Illumina, San Diego, CA), generating 250 bp paired end reads. The paired reads were merged using FLASH (V1.2.7,http://ccb.jhu.edu/software/FLASH/). Quality filtering on the raw tags were performed under specific filtering conditions to obtain the high-quality clean tags according to the QIIME (V1.7.0, http://qiime.org/index.html) quality-controlled process. Chimeric sequences were removed using UCHIME algorithm (UCHIME Algorithm, http://www.drive5.com/usearch/manual/uchime_algo.html) against the reference database (Gold database, http://drive5.com/uchime/ uchime_download.html)0.19 Reads were clustered into operational taxonomic units (OTUs) using Uparse software (Uparse v7.0.1001, http://drive5.com/uparse/). Sequences with ≥97% similarity were assigned to the same OTUs. Representative sequence for each OTU was screened for further annotation. For each representative sequence, the Green Gene Database (http://greengenes.lbl. gov/cgi-bin/nph-index.cgi) was used based on RDP classifier (Version 2.2, http://sourceforge.net/projects/rdp-classifier/) algorithmto annotate taxonomic information. Alpha diversity is applied in analyzing complexity of species diversity for a sample through 6 indices, including Observed-species, Chao1, Shannon, Simpson, ACE, Good-coverage. All these indices in our samples were calculated with QIIME (Version 1.7.0) and displayed with R software (Version 2.15.3). Beta diversity analysis was used to evaluate differences of samples in species complexity, Beta diversity on both weighted and unweighted unifrac were calculated by QIIME software (Version 1.7.0). Cluster analysis was preceded by PCA, which was applied to reduce the dimension of the original variables using the FactoMineR package and ggplot2 package in R software (Version 2.15.3).

## Histology

Paraffin sections (5 μm) of ileum were attached to poly-L-lysine-coated glass slides. After overnight incubation at 37 °C, slides were de-waxed and hydrated step-wise using 100% xylene followed by several solutions of distilled water containing decreasing amounts of ethanol. Sections were stained with Alcian Blue Periodic acid Schiff (AB-PAS) Stain Kit (Solarbio, China)^35,36^. Mucous cells in small intestinal epithelium were counted (counting the number of cells in 5 adjacent villi per quadrant/4 quadrants/per section/2 sections per animal/7 animals per group) using Motic EasyScanner and DSAssistant software (Changsha Central Hospital, China).

### Statistical analysis

Data on weight gained and the percentage of classified sequence reads were expressed as mean ± standard deviation. Student’s t-test, Mann-Whitney test and one-way ANOVA were used for statistical analysis, and Wilcox was used to elevate α and β diversity. Spearman’s test was applied to analyze the relationship between weight gain and gut microbiota. P value < 0.05 was considered to be significantly different.

### Ethical approval and informed consent

All animal protocols and experiments were approved by the institutional animal care committee of 2ndxiangya hospital at Central South University, and all the methods were carried out in accordance with the relevant guidelines and regulations.

## Supplementary information

Supplementary text and figures

## Data Availability

All data generated or analysed during this study are included in this published article (and its Supplementary Information files).
