# Supplementary material for: Effects of Different Diets on Microbiota in The Small Intestine Mucus and Weight Regulation in Rats
Source: Sci Rep. 2019 Jun 11;9:8500. doi: 10.1038/s41598-019-44994-7 (PMC6560036; doi:10.1038/s41598-019-44994-7)
Supplement: Supplementary file 1 — Supplementary text and figures [file 41598_2019_44994_MOESM1_ESM.pdf]

Title: Effects of Different Diets on Microbiota in The Small Intestine Mucus and Weight Regulation in Rats

Authors and affiliations:

Yu Meng [1], Xiaojun Li [1], Jie Zhang [1], Chunlian Wang[1] and Fanggen Lu[1]*

[1] Departments of Gastroenterology, The Second Xiangya Hospital of Central South University, Changsha, Hunan, P.R. China; mengyu001@csu.edu.cn

***** Correspondence: fanggenlu@csu.edu.cn; Tel.: +86-138-0842-3923

SUPPLEMENTAL FIGURE LEGENDS

Figure S1. Community bar blots of the relative abundance at class level (A), order level (B) and family level (C) in SIM and fecal samples of each group. D. Taxa tree illustrated the origin of gut microbiota and the proportion of gut microbiota in each group.

Figure S2. Heatmaps of changes in bacterial abundance at different levels in SIM samples and fecal samples of each group. A. At class level, B. At order level, C. At family level), and at genus level (D).

Figure S3. Study design. The rats were fed with STD, HFD, HSD and HPD, respectively, and were sacrificed after 12 weeks. SIM and fecal samples were collected for microbiota analysis and the ileum tissues were collected for histological staining.

Figure S1

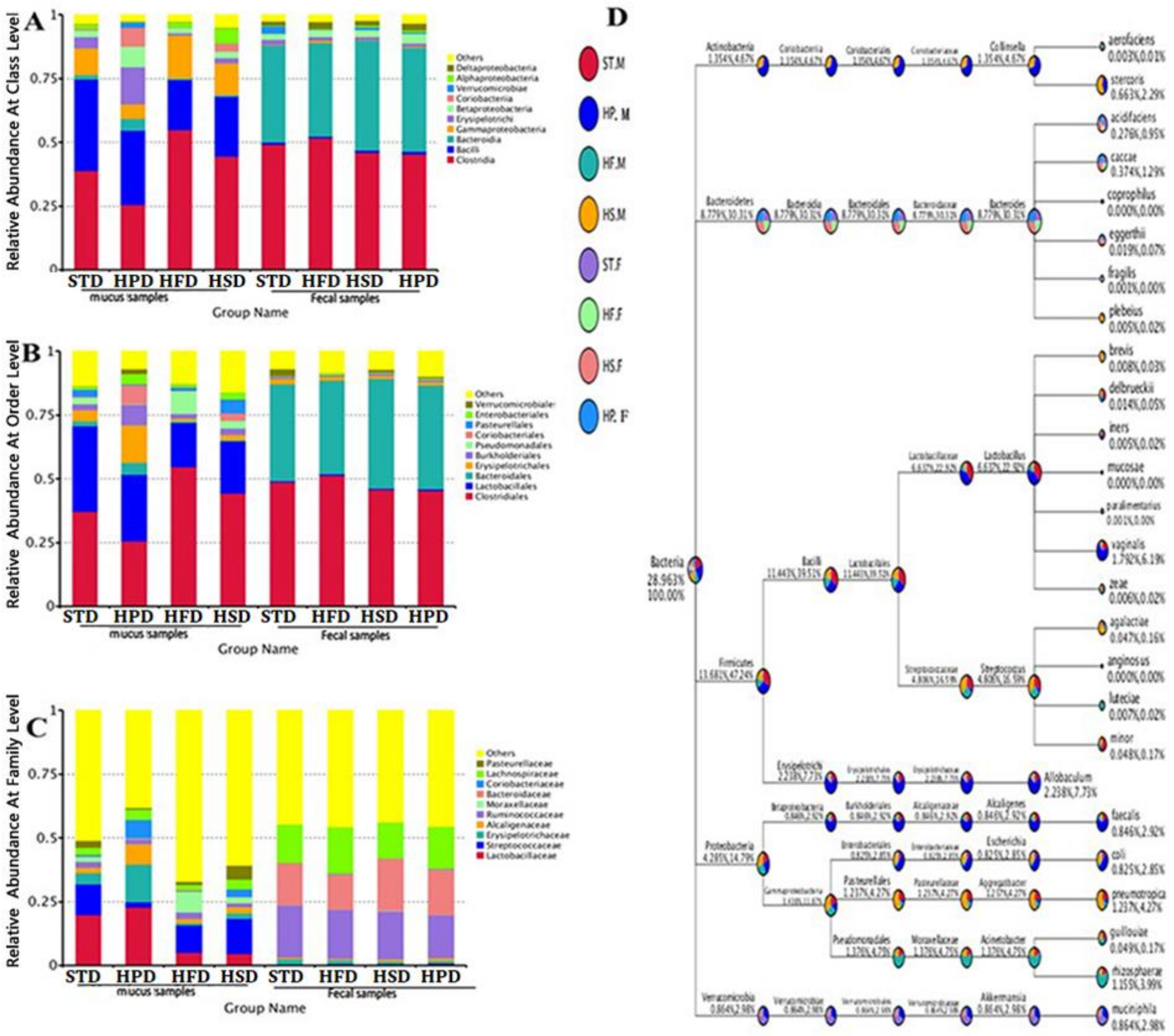
A
B
C
D
Relative Abundance At Class Level
Relative Abundance At Order Level
Relative Abundance At Family Level
STD HPD HFD HSD STD HFD HSD HPD
mucus samples
Fecal samples
Group Name
ST.M
HP. M
HF.M
HS.M
ST.F
HF.F
HS.F
HP. F
Bacteria
28.963%
100.00%

Figure S2

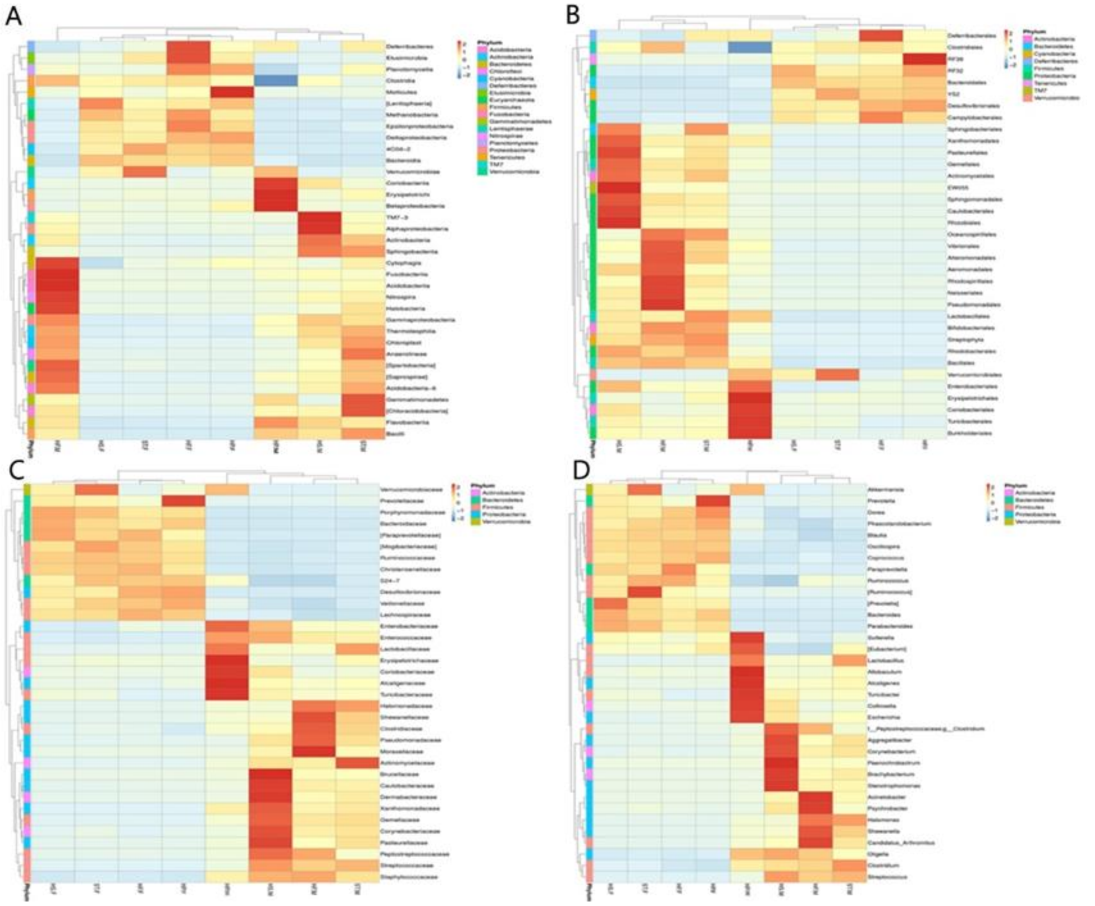

Figure S3

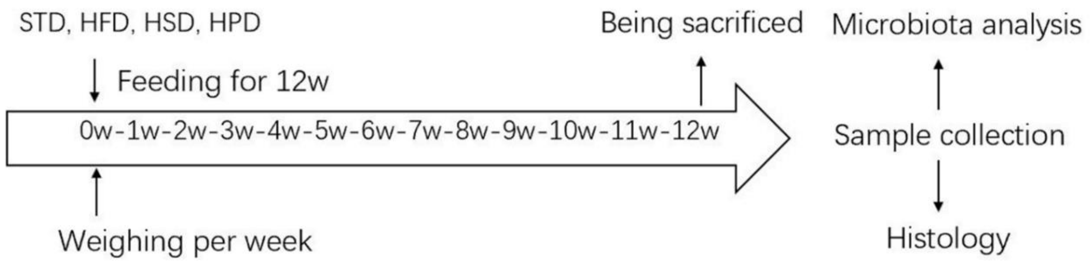
STD, HFD, HSD, HPD
Feeding for 12w
0w-1w-2w-3w-4w-5w-6w-7w-8w-9w-10w-11w-12w
Weighing per week
Being sacrificed
Microbiota analysis
Sample collection
Histology
